# MSLP: mRNA subcellular localization predictor based on machine learning techniques

**DOI:** 10.1186/s12859-023-05232-0

**Published:** 2023-03-22

**Authors:** Saleh Musleh, Mohammad Tariqul Islam, Rizwan Qureshi, Nehad M. Alajez, Tanvir Alam

**Affiliations:** 1 College of Science and Engineering, Hamad Bin Khalifa University, Doha, Qatar; 2 Computer Science Department, Southern Connecticut State University, New Haven, CT USA; 3 Translational Cancer and Immunity Center (TCIC), Qatar Biomedical Research Institute (QBRI), Hamad Bin Khalifa University, Doha, Qatar; 4 College of Health and Life Sciences, Hamad Bin Khalifa University, Doha, Qatar

**Keywords:** RNA, mRNA, Machine learning, Sequence analysis, Localization prediction, Subcellular localization

## Abstract

**Background:**

Subcellular localization of messenger RNA (mRNAs) plays a pivotal role in the regulation of gene expression, cell migration as well as in cellular adaptation. Experiment techniques for pinpointing the subcellular localization of mRNAs are laborious, time-consuming and expensive. Therefore, in silico approaches for this purpose are attaining great attention in the RNA community.

**Methods:**

In this article, we propose MSLP, a machine learning-based method to predict the subcellular localization of mRNA. We propose a novel combination of four types of features representing k-mer, pseudo k-tuple nucleotide composition (PseKNC), physicochemical properties of nucleotides, and 3D representation of sequences based on Z-curve transformation to feed into machine learning algorithm to predict the subcellular localization of mRNAs.

**Results:**

Considering the combination of the above-mentioned features, ennsemble-based models achieved state-of-the-art results in mRNA subcellular localization prediction tasks for multiple benchmark datasets. We evaluated the performance of our method  in ten subcellular locations, covering cytoplasm, nucleus, endoplasmic reticulum (ER), extracellular region (ExR), mitochondria, cytosol, pseudopodium, posterior, exosome, and the ribosome. Ablation study highlighted k-mer and PseKNC to be more dominant than other features for predicting cytoplasm, nucleus, and ER localizations. On the other hand, physicochemical properties and Z-curve based features contributed the most to ExR and mitochondria detection. SHAP-based analysis revealed the relative importance of features to provide better insights into the proposed approach.

**Availability:**

We have implemented a Docker container and API for end users to run their sequences on our model. Datasets, the code of API and the Docker are shared for the community in GitHub at: https://github.com/smusleh/MSLP.

**Supplementary Information:**

The online version contains supplementary material available at 10.1186/s12859-023-05232-0.

## Introduction

**Fig. 1:**
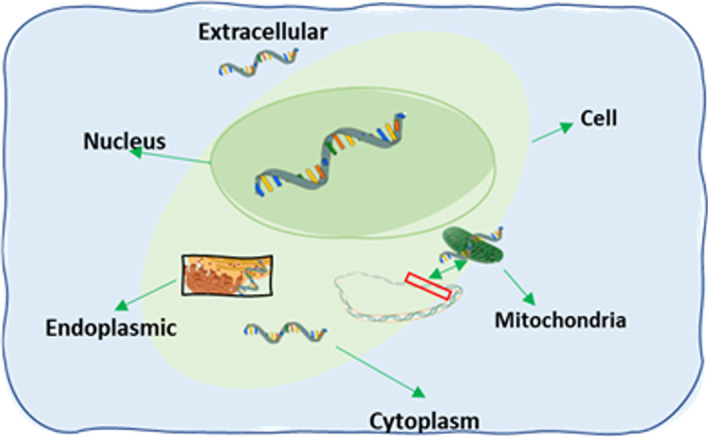
Symbolic Diagram of a typical animal cell with five subcellular localization: nucleus, mitochondria, cytoplasm, ER and ExR

Messenger RNA (mRNA) is a single-strand RNA molecule which is complementary to one of the DNA strands of a genome. In the transcription process, these RNAs are spliced, capped, polyadenylated to move between different nucleus parts and further to be exported to cytoplasm and secreted into extracellular regions [1]. With the discovery of the asymmetric distribution of $\beta$-actin mRNA in ascidian embryos and eggs, Jeffery et al. laid the foundation for mRNA subcellular localization studies [2]. Later the non-random distribution of mRNAs in cytoplasm for cytoskeletal proteins hints at a mechanism for quantifying its concentration [3]. Since then localization of mRNAs has been discovered to be linked to varieties of cellular processes and their regulatory roles in cells [4]. Localization of mRNAs also plays a vital role in spatio-temporal regulation of gene expression as well as development process in the cell, including cell migration and cellular adaptation [5, 6]. Localization of mRNAs also facilitates the subcellular localization of proteins to maintain cell polarity, synaptic plasticity responsible for long-lasting memory, assembly of protein complexes and regulation of differential translation [7–10]. Moreover, deregulation of mRNA localization may cause multiple genetic disorders and cancer as well [11]. Figure 1 shows a schematic diagram of mRNA localization at subcellular level. With the advancement of experiment techniques, subcellular localization of many RNAs have been detected so far [12]. Among the existing techniques, RNA fluorescent in situ hybridization (RNA-FISH) is one of the reliable experiment techniques for mRNA localization identification, but it is slow and laborious and is limited to specific tissues [13, 14]. Recently high throughput techniques such as APEX-RIP and CeFra-seq are also proposed for determining the subcellular localization of RNA. But the data generated by APEX-RIP [15] or CeFra-seq [16] are noisy and might not be highly accurate [1]. Moreover, all the experiment techniques for determining the localization of mRNA are expensive, time-consuming and hence, the development of in silico methods based on machine learning (ML) modeling is gaining momentum in the RNA society [17].

RNATracker was the first computational method developed based on recurrent neural networks (RNN) to predict mRNA subcellular localization [1]. The authors used the sequence as well as the secondary structure of mRNA to predict the subcellular localization. The authors encoded the mRNA sequence and the predicted secondary structure as 4-bit and 6-bit one hot encoding, respectively. Sequences longer than 4000 nt were truncated at $5^{\prime }$ end and shorter sequences were padded with zeros. Then the embedding was fed into convolutional neural network (CNN) coupled with bidirectional long short-term memory (BiLSTM) network with attention mechanism. The authors used two benchmark datasets from CeFra-Seq and APEX-RIP for the prediction. It is pertinent to emphasize that data from CeFra-Seq and APEX-RIP might not be very accurate and inherently noisy [1].

Zhang et al. developed the iLoc-mRNA method for human mRNA subcellular localization prediction [18]. The authors used k-mer (k=9) for generating features from mRNA sequence and subsequently ANOVA technique combined with binomial distribution was used to select a subset of features from k-mer. Finally a support vector machine (SVM) with radial basis function (RBF) was used to predict the mRNA subcellular localization. It is important to emphasize that the authors combined mRNAs from multiple locations into a single custom location i.e., C1, C4 which might not reflect the actual localization at cellular level [19]. For example the authors combined mRNAs from nucleus, exosome, dendrite and mitochondria into class C4; mRNAs from cytosol and cytoplasm into class C1. So, the custom classes i.e., C1,C4 are not representing actual biological locations.

Recently Garg et al. proposed mRNALoc [19] to predict mRNA subcellular localization in five locations, namely cytoplasm, nucleus, ER, ExR, and mitochondria considering dataset from the RNALocate database [20]. From the input mRNA sequences, the authors generated pseudo k-tuple nucleotide composition (PseKNC) of different k (=2 to 5) to generate features. The features were then fed into an SVM based model to predict mRNA subcellular localization. The authors developed five different SVM models for five locations and based on the prediction score, the localization of mRNA was determined.

Meher et al. developed mLoc-mRNA, a random forest (RF) based method for mRNA subcellular location prediction [21]. The authors used nine different locations from the RNALocate database [20]. From the sequence of mRNA the authors generated k-mer (k=1 to 6) based features to encode the sequence and an elastic net was used to select a subset of features. Finally the selected features were fed into RF based nine classifiers for predicting nine locations.

Li et al. proposed SubLocEP [22], a two-layer prediction model for predicting the location of sequence samples. In this study, both the training and testing datasets were created using the RNALocate 2.0 dataset. The team has extracted nine different feature categories in this study to build the single-layer initial model. Weighting the sequence-based physicochemical properties at 3:2 led to the final two-layer model. The models were designed to predict mRNA localizations more accurately and to generalize to new datasets, according to the researchers. The findings of the five-fold cross-validation experiment indicate that the single-layer sequence-based LightGBM models have an average accuracy of 65%. The performance of the single-layer physiochemical property models was marginally higher at 65.9%. The SubLocEP achieved 66% accuracy and better performance in the two-layer model. The independent datasets’ one and two accuracy results ranged from 48.68 to 60.10%.

Qiang Tang et al. developed mRNALocater [23] to predict mRNA subcellular localization by incorporating PseKNC (k=2 to 6) and PseEIIP. Features having a correlation factor of more than 85% were filtered using a two-step feature optimization method. Sequential forward search (SFS) methodology was then utilized to identify the best feature subsets. The team has used the LightGBM model to determine the feature relevance. The LightGBM model performed well at predicting the location in the ER and mitochondria. The CatBoost (CatB) model had a great performance at predicting the location in the extracellular region, with an accur of 86.16%. The XGBoost (XGB) model had the best performance at identifying the locations in the cytoplasm and nucleus, with an accuracy of 63.23% and 69.83%, respectively. Summarily, these findings show that the boosting based models are complementary to predict the localization of mRNA from different organelles and each model has its own advantages over others [23]. Table 1 summarizes the literature that considered ML based approach for the mRNA localization prediction problem.

**Table 1:** Summary of previous articles focusing on machine-learning based mRNA subcellular localization prediction

References	Year	Subcelular localizaiton	#location	Proposed model	Features/encoding
RNATracker [1]	2019	CeFra-Seq (Cytosol,Nuclear, Membrane, Insoluble); APEX-RIP (Cytosol, Nuclear, ER, Mitochondria)	4	CNN, BLSTM, Attention mechanism	One hot encoding of sequence
iLoc-mRNA [18]	2020	4 custom locations : C1, C2, C3, C4 were designed from nine subcellular locations (Cytosol, Cytoplasm, Ribosome, ER, Nucleus, Exosome, Mitochondria, Dendrite)	4	SVM	k-mer (k=9)
mRNALoc [19]	2020	Cytoplasm, Nucleus, ER, ExR, Mitochondria	5	SVM	Pse-KNC (k=2,..5)
mLoc-mRNA [21]	2021	Cytoplasm, Nucleus, ER, Mitochondria, Cytosol, Pseudopodium, Posterior, Ribosome, Exosome	9	RF	k-mer (k=1…6)
SubLocEP [22]	2021	Cytoplasm, Nucleus, ER, ExR, Mitochondria	5	LightGBM	k-mer (k=2,3), parallel correlation of PseKNC (k=2,3), series correlation of PseKNC (k=2,3), physicochemical properties (PseEIIP)
mRNALoacter [23]	2021	Cytoplasm, Nucleus, ER, ExR, Mitochondria	5	LightGBM, XGBoost, CatBoost	PseKNC (k=2,..,6), physicochemical properties (PseEIIP)
MSLP (our method)	2022	Cytoplasm, Nucleus, ER, ExR, Mitochondria, Cytosol, Pseudopodium, Posterior, Ribosome, Exosome	10	CatBoost	k-mer (k=2,..,5), PseKNC (k=2,…,5), physicochemical properties PseEIIP, DPCP, TPCP, Z-curve

From the discussion above, it is pertinent to highlight that ML-based methods can be useful for this important research problem considering its high accuracy as well as minimal cost. This motivated us to develop a new computational method MSLP (mRNA Subcellular Localization Predictor) for predicting the subcellular localization of mRNAs. The contribution of this work can be summarized as follows:

1. We proposed a novel combination of features to represent mRNA using k-mer, pseudo nucleotide composition, physicochemical properties, and 3D representation of sequence in Z-curve transformation to predict mRNA subcellular localization. The novel combination of features showed better performance compared to the existing methods for the same purpose.
2. We considered multiple benchmark datasets for mRNA subcellular localization prediction task for ten locations, covering the highest number of subcellular locations in literature and outperformed existing methods for the same purpose in the majority of localization from all datasets.
3. We showed that different subsets of features are suitable for localizing mRNAs at different locations, rather than a canonical set of features. Specifically, we showed that k-mer and PseKNC were more dominant than other features for predicting cytoplasm, nucleus, and ER. But physicochemical properties and Z-curve based features were considered as the dominant feature set for ExR and mitochondria localization prediction.
4. We have implemented a Docker container and API for end users to run their sequences on the proposed model. The source code and Docker is made available for community users.

## Materials and methods

**Fig. 2:**
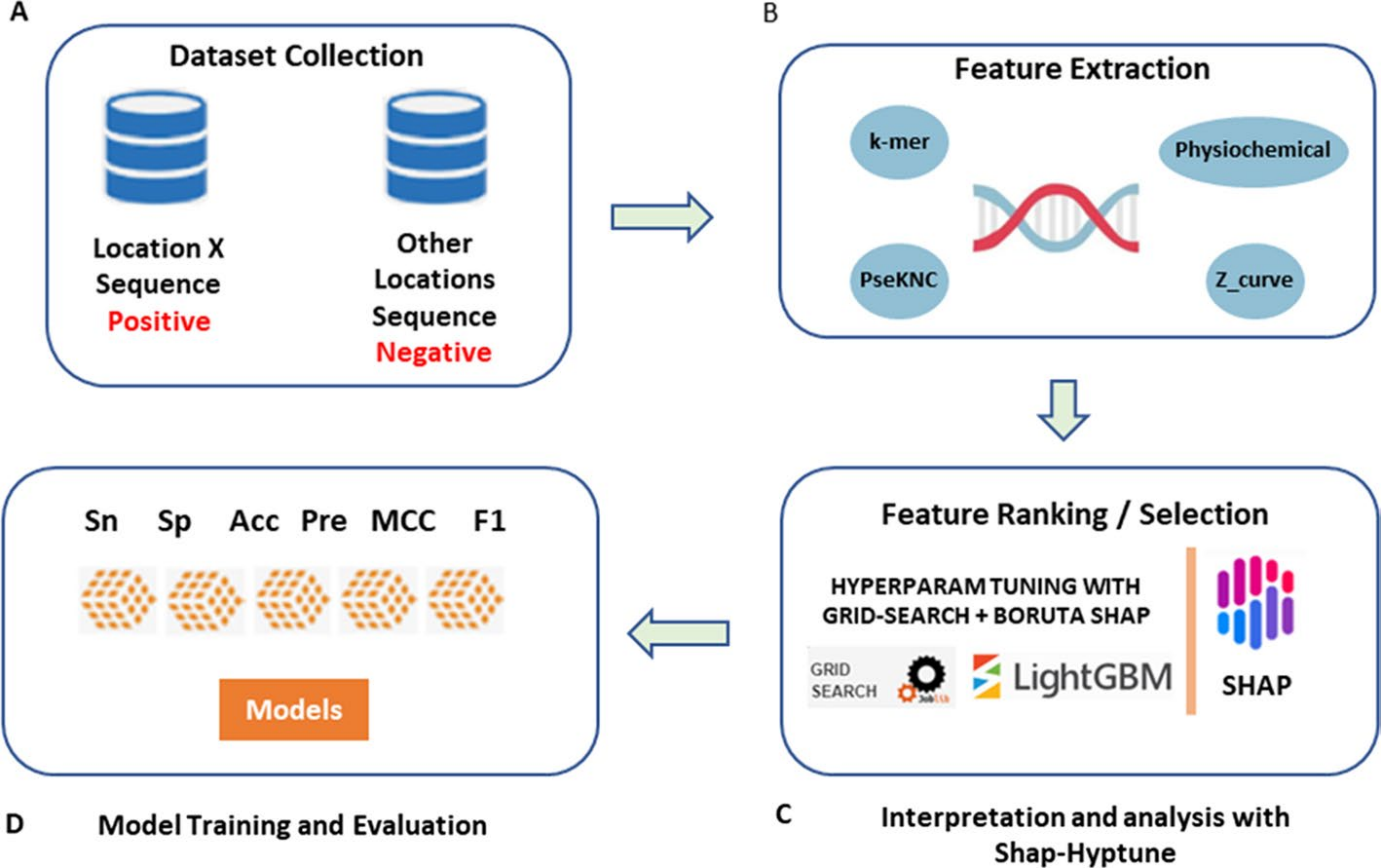
Overall computing pipeline for the proposed mRNA subcellular Localization Predictor (MSLP). **a** Dataset collection from multiple sources. **b** Feature engineering for the proposed feature. **c** Feature subset selection from the pool of features. **d** Machine learning model development and validation

To predict the subcellular localization of mRNA, we gathered the largest collection of mRNA sequences from ten cellular locations that are mentioned in the literature. After the data collection steps had completed, we generated numerical features from the input sequences and their physicochemical properties. We then used these features to build different classifiers in order to predict the mRNA subcellular location from the given sequences. Figure 2 highlights the computational workflow of the MSLP method.

### Dataset collection and processing

We experimented with two benchmark dataset in this work. The first of these uses the five of the most commonly used locations from existing literature as the class labels that was proposed in mRNALoc [19]. This configuration was necessary to be able to compare against the rest of the prominent research works. The second dataset uses the five other locations proposed recently in mLoc-mRNA [21]. This dataset is also important since it uses locations that were introduced for the first time in the cited work. We describe the dataset collection and feature formation for both configurations below.

The first dataset was collected from mRNALoc [19], where the authors considered the data of mRNA subcellular localization from RNALocate v2.0 [12]. RNALocate is widely accepted as a repository for subcellular localization information of RNAs as it considers multiple sources of experimentally validated information followed by manual curation [12]. The downloaded mRNA sequences belongs to both single and multiple subcellular locations. For our study, we considered only mRNA sequences that were confined to a single subcellular location and discarded mRNAs located in multiple subcellular locations, an approach also used in [19] and [22]. It is important to emphasize that the majority of the existing methods for mRNA subcellular localization prediction considered five locations (see Table 1). Therefore, we considered the same five locations for training and validation of MSLP. Following the same pipeline prescribed in [19] and [22], we considered the non-redundant (NCBI BLASTCLUST tool with the option ”-S 40 and -L 0.70”) dataset of mRNA from five sublocations: 350 in mitochondria, 710 in extracellular region (ExR), 1185 in endoplasmic reticulum (ER), 4855 in nucleus, and 5310 in cytoplasm. To avoid overestimating the performance of MSLP in comparing against the same from the other methods, we used an independent test dataset (TEST-01) that was not used during the training and validation of ML models. TEST-01 contains 1066, 976, 241, 145, 71 sequences of mRNA localized in and cytoplasm, nucleus, ER, ExR, and mitochondria respectively.

Moreover, we collected the second dataset from mLoc-mRNA [21] where the authors considered nine subcellular locations of mRNAs, namely cytoplasm, nucleus, ER, mitochondria, posterior, pseudopodium, exosome, ribosome, and cytosol. As the article covers five new subcellular location namely cytosol, exosome, ribosome, posterior, and pseudopodium we considered these additional five locations for training and validation as well. For these additional five locations, validation was done using two independent datasets (IDS-I and IDS-II) for these new five subcellular locations. For these five subcellular locations we had 1798, 843, 1838, 187, 216 sequences for training from cytosol, exosome, ribosome, posterior, and pseudopodium, respectively. For these five subcellular locations we had 360, 140, 306, 31, 36 sequences in IDS-01 and 1037, 185, 789, 121, 79 sequences in IDS-02 from cytosol, exosome, ribosome, posterior, and pseudopodium, respectively.

**Fig. 3:**
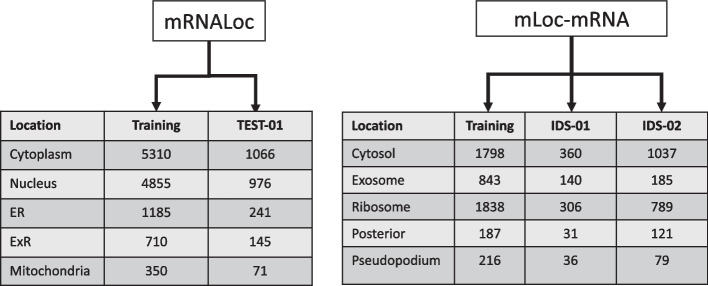
Overall statistics of the number of sequence used from ten different locations

In summary, in this article we covered ten different locations to cover the highest number of cellular sublocations of mRNA in the literature till to date. Figure 3 summarizes the overall statistics of the datasets we used.

### Preparation of the positive and negative datasets

For a particular subcellular location, we considered the DNA sequence of all mRNAs that are coming from that particular location as the positive set for the ML model. We then randomly selected the sequences from the other locations as the negative set for ML model. This yields a positive and negative dataset pair for each subcellular localization prediction model.

### Feature extraction

In this study, we extracted four types of DNA features. These include K-mer, Pseudo K-tuple Nucleotide Composition (PseKNC), Physicochemical Properties of mRNA transcripts (PseEIIP, DPCP, and TPCP), and Z curve parameters for phase-specific and phase-independent trinucleotide frequencies. The sizes of the feature vectors for each were 1360, 1370, 64, 2368, 768, 48, and 144, respectively.The following subsections detail each of these feature extraction processes.

#### K-mer related features

For each DNA sequence of the corresponding mRNA, we calculated the frequencies of mono-, di-, tri-, quad-, and penta-consecutive nucleotides (i.e., k-mers with k=2,3,4,5) in the whole transcript body. We then normalized the k-mer count by the sequence length. These two steps can be summarized using the equation:

1 $$\begin{aligned} Kmer_{i} = \frac{C_i}{L}, k=2,3,4, and \ 5 \end{aligned}$$

where, ${C_i}$ is the k-mer count in the transcript and *L* represents the length of the transcript. This generated a 1360-dimensional feature vector which is a concatenation of 16-, 64-, 256-, and 1024-dimensional vectors from di-, tri-, quad-, and penta-mer consecutive nucleotides, respectively. This feature vector is then used for representing the k-mer features in each input sequence.

#### Pseudo K-tuple nucleotide composition (PseKNC)

The PseKNC of sequence reflects the nucleotide-order effects in DNA sequence [24, 25]. This order-specific information is preserved through the physicochemical properties of the constituent nucleotides. The feature vector is of size $(4^k+\lambda )$ where k represents the length of k-mer, and $\lambda$ represents the highest counted rank of the correlation along the sequence. In our study we considered $k=2,3,4,5$ and $(\lambda =10)$ to generate 16, 64, 256, 1024, and 10 features and combined them to generate a 1370-dimensional feature vector for the corresponding DNA sequence of an mRNA.

#### Physicochemical properties of mRNA genes

For capturing the physicochemical properties of nucleotides we used three types of features, namely Pseudo Electron-Ion Interaction Pseudopotentials (Pse-EIIP) of trinucleotide, dinucleotide physicochemical properties (DPCP), and trinucleotide physicochemical properties (TPCP), the details of which can be found below. For generating these features from the DNA sequence of the corresponding mRNA, we used the iLearnPlus [26] tool.

- Pseudo Electron-ion interaction pseudopotentials (PseEIIP): EIIP represents the energy of delocalized electrons in nucleotides or amino acids as proposed in [27, 28]. As an illustration of how to generate the EIIP indicator sequence, consider the following EIIP values of nucleotides: *A*, *C*, *G*, and *T* as 0.1260, 0.1340, 0.0806, and 0.1335, respectively. If we substitute the *EIIP* values for *A*, *C*, *G* and *T* in a DNA string *X*[*n*], we get a numerical sequence that represents the distribution of the energies of the free electrons along the input sequence. This sequence is known as the *EIIP* indicator sequence of *X*[*n*]. For example, if $X[n]=ATAGCATCA$, then using the above nucleotides *EIIP* values, we get
  2 $$\begin{aligned} \begin{aligned} X[n]=&\,[0.1260,0.1335,0.1260,0.0806, \\&0.1340,0.1260,0.1335,0.1340,0.1260] \\ \end{aligned} \end{aligned}$$
  Now to calculate the *PseEIIP*, let $EIIP_A$, $EIIP_C$, $EIIP_G$, and $EIIP_T$ denote the *EIIP* values of nucleotides *A*, *C*, *G* and *T*, respectively. Then, the vector of weighted *EIIP* values of trinucleotides in each sequence can be formulated as:
  3 $$\begin{aligned} \begin{aligned} V=&\,[EIIP_{AAA} \cdot f_{AAA}, EIIP_{AAA} \cdot f_{AAC},..., \\&EIIP_{TTT} \cdot f_{TTT}]\\ \end{aligned} \end{aligned}$$
  Here, $f_{xyz}$ is the normalized frequency of the *i*th trinucleotide, where *x*, *y*, *z* $\epsilon$ [*A*, *C*, *G*, *T*]. $EIIP_{xyz}$ = $EIIP_x$ + $EIIP_y$ + $EIIP_z$ represents the *EIIP* value of a single trinucleotide. The dimension of the generated vector for each DNA sequence of mRNA was 64.
- Dinucleotide physicochemical properties (DPCP): The DPCP descriptor can be defined as:
  4 $$\begin{aligned} \begin{aligned} V =&\, DPCP_{AA} \times f_{AA}, DPCP_{AC} \times f_{AC},...,\\&DPCP_{TT} \times f_{TT}\\ \end{aligned} \end{aligned}$$
  Here, $f_{xy}$ is the normalized frequency of the *i*th dinucleotide and *x*, *y* $\epsilon$ [*A*, *C*, *G*, *T*].. $DPCP_{xy}$ is one of the 148 physicochemical properties for DNA dinucleotides described in [26] and listed in Additional file 1: File S1. The dimension of generated feature vector *V* was 2368 $(148\times 16)$.
- Trinucleotide physicochemical properties (TPCP): The TPCP descriptor can be defined as:
  5 $$\begin{aligned} \begin{aligned} V=&\,[TPCP_{AAA}.f_{AAA}, TPCP_{AAA}.f_{AAC},..., \\&DPCP_{TTT}.f_{TTT}]\\ \end{aligned} \end{aligned}$$
  Here, $f_{xyz}$ is the normalized frequency of the *i*th trinucleotide and *x*, *y*, *z* $\epsilon$ [*A*, *C*, *G*, *T*].. $TPCP_{xyz}$ is one of the twelve physicochemical properties of a trinucleotide listed in Additional file 1: File S1. The twelve physicochemical properties for DNA trinucleotides are named as “Bendability (DNase)”, “Bendability (consensus)”, “Consensus rigid”, “Consensus roll”,“DNase I”, “DNase I rigid”, “Nucleosome”, “Nucleosome Rigid”, “Nucleosome positioning”, “MW Daltons”, “MW-kg”, “Trinucleotide GC content” in [26] and listed in Additional file 1: File S1. The dimension of the generated feature vector *V* was 768 ($12\times 64$).

#### Z-curve parameters for phase-specific and phase-independent trinucleotide frequencies

The Z-curve theory entails a geometrical approach to represent a genome sequence in 3-D space [29, 30]. The frequency of nucleotides A, C, G and T or their combinations (k-mer) occurring in the sequence or open reading frame are transformed into 3D space based on Z-transform [31], which is used to derive the equation of the Z-curve. The Z-curve has been successfully applied in the identification of protein-coding genes, finding new genes in eukaryotic organisms, CG content variation, etc. [30]. We considered the following representation of trinucleotides in terms of the Z-curve for feature engineering.

1. Phase-independent tri-nucleotides frequency: This can be represented using Z-curve parameters by a 48-bit descriptor as follows:
  6 $$\begin{aligned} {\left\{ \begin{array}{ll} x_{XY}=[(p(XYA)+p(XYG))-(p(XYC)+p(XYT)]\\ y_{XY}=[(p(XYA)+p(XYC))-(p(XYG)+p(XYT)]\\ z_{XY}=[(p(XYA)+p(XYT))-(p(XYC)+p(XYG)]\\ \end{array}\right. } \end{aligned}$$
  where the normalized frequency of trinucleotides *XYA*, *XYC*, *XYG*, *XYT* are represented by *p*(*XYA*), *p*(*XYC*), *p*(*XYG*), *p*(*XYT*) respectively. The dimension of the feature matrix is 48.
2. Phase-specific tri-nucleotide frequency: This can be represented using Z-curve parameters by a 144-bit descriptor as follows:
  7 $$\begin{aligned} {\left\{ \begin{array}{ll} x^{k}_{XY}=[(p^{k}(XYA)+p^{k}(XYG))-(p^{k}(XYC)+p{k}(XYT)]\\ y^{k}_{XY}=[(p^{k}(XYA)+p^{k}(XYC))-(p^{k}(XYG)+p^{k}(XYT)]\\ z^{k}_{XY}=[(p^{k}(XYA)+p^{k}(XYT))-(p^{k}(XYC)+p^{k}(XYG)]\\ \end{array}\right. } \end{aligned}$$
  where *k* represents the position of nucleotide(s) at the first, second, or third position of potential codons. The normalized frequency of trinucleotides *XYA*, *XYC*, *XYG*, *XYT* at different positions were represented by $p^{k}(XYA)$, $p^k(XYC)$, $p^k(XYG)$, $p^k(XYT)$ respectively. The dimension of the feature matrix is 144. The name of all features are provided in Additional file 1: File S2.

### Development of classification models

This section describes the development of the classification models for subcellular mRNA localization. We first provide the reasoning for using One-versus-Rest classifiers in our proposed method. We then present the candidate models, and lastly, explain the model selection process.

#### One-versus-rest (OvR) approach for multi-class supervised learning

The development of classification models for the task at hand needed to consider the multi-class nature of the problem. We decided to employ multiple one versus rest (OvR) binary classifiers to accomplish the task. Binary classification is a task where samples are assigned precisely to one of two classes. On the other hand, multi-class classification is a task where samples are assigned to exactly one of many (more than two) classes. The multi-class classification tasks can either be approached as-is or can be simplified into multiple binary classification problems. For the former, we need to consider building one classifier for all class labels. For example, if we have five different class labels, the model will provide the probability of each class such that the summation of all probabilities is equal to one. One of the major limitations of this approach is that if we have many classes, usually the performance of the model drops down. It has been shown [32] that One-versus-Rest is a better technique for multi-class classification problems. The latter can be solved either using One versus rest (OvR) where a binary classifier is built for each class considering one class as the positive and the rest of the samples as the negative class, or one versus one (OvO) where a binary classifier is built for each pair of classes.

#### Candidate models

We experimented with multiple ML classifiers, namely Decision Tree (DT), Gaussian Naive Bayes (GNB), Support Vector Classifier (SVC) with radial basis function (rbf) kernel, Random Forest (RF), CatBoost (CatB), and XGBoost (XGB) in Python. It is worthy to mention that for ExR and Mitochondria, we use 1:2 and 1:3 ratio of positive:negative samples, respectively to train the model. For other locations, we used a 1:1 ratio of positive:negative dataset. Hyperparameters were optimized using GridSearchCV and early stopping from the Scikit-Learn package in Python.

#### Model selection for individual subcellular localization predictor

**Fig. 4:**
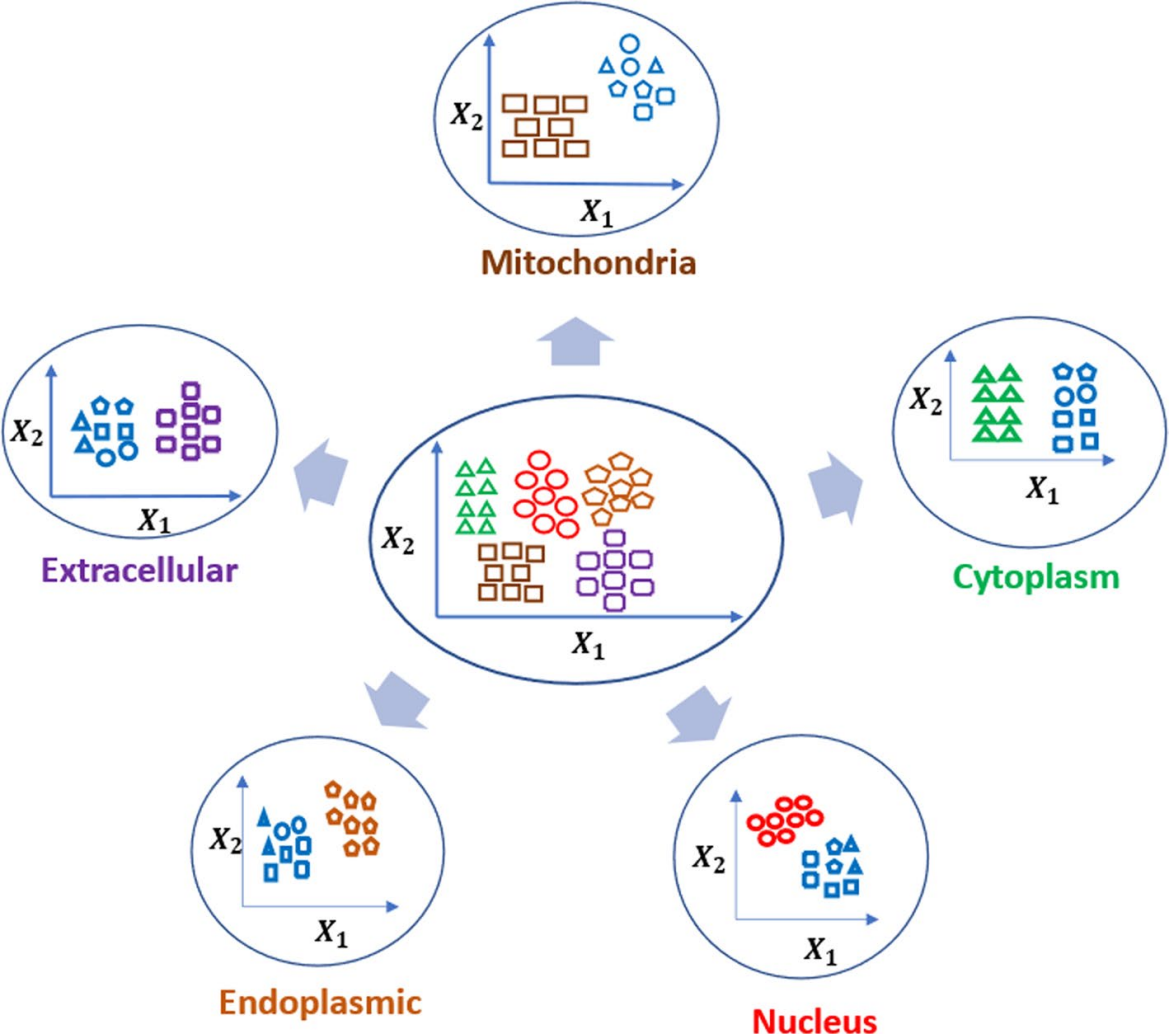
One versus Rest (OvR) approach for classifying mRNA subcellular localization. Middle panel highlights the original dataset, the surrounding panel highlights the OvR approach adapted

We experimented with five OvR models for each subcellular location: Cytoplasm, Nucleus, ER, ExR, and Mitochondria. We built binary classifiers for cytoplasm versus rest, ”Nucleus” versus rest, ER versus rest, ExR versus rest, and mitochondria versus rest. Figure 4 shows a visual representation of the OvR strategy for mRNA localization problems. Based on the findings from the ablation study on feature combinations, we selected one inference model for each location with a particular set of features which resulted in the highest performance for localizing to the corresponding location. The models corresponding to Cytoplasm, Nucleus, and ER use K-mer and PseKNC while the Mitochondria and ExR models use the Physiochemical properties and Z-Curve features.

Performance evaluation on the test sets and inference is carried out as follows. For each test (or unknown) example, we use (i) K-mer and PseKNC feature values to obtain the localization scores $(l_{cyto}, l_{ER}, l_{Nuc})$ for Cytoplasm, ER, and Nucleus from the respective models, and (ii) Physicochemical properties and Z-Curve values to obtain localization scores $(l_{ExR}, l_{Mito})$ for ExR and Mitochondria from the corresponding models. We then assign the input sequence to the location corresponding to the highest score $max\{l_{cyto}, l_{ER}, l_{Nuc}, l_{ExR}, l_{Mito}\}$.

### Performance evaluation of the models

Performance evaluation for ML model is one of the critical steps in building an effective model as it involves the selection of the best model and measuring its generalization performance on an unseen section of the dataset, both of which are prone to data leakage leading to performance overestimation. For the latter, we first had set aside 20% of the datasets for purely test purposes, so that data leakage does not occur between the model selection and the generalization stages. For the model training and selection stage, we used five-fold cross-validation (CV) to obtain consistent results. For each fold in a five-fold CV setting, 80% of the remaining data was used for training and the other 20% for validation of the model.

Different performance evaluation metrics provide means to assess the model’s performance and quality. These performance metrics show how well the model has performed for the given data. We used the following metrics for evaluation the models:

8 $$\begin{aligned} Sensitivity (recall)= & {} \frac{tp}{tp+fn} \end{aligned}$$

9 $$\begin{aligned} Specificity= & {} \frac{tn}{fp+tn} \end{aligned}$$

10 $$\begin{aligned} Accuracy= & {} \frac{tp+tn}{tp+fn+fp+tn} \end{aligned}$$

11 $$\begin{aligned} Precision= & {} \frac{tp}{tp+fp} \end{aligned}$$

12 $$\begin{aligned} F1 Score= & {} \frac{2*Precision*Recall}{Precision+recall} \end{aligned}$$

13 $$\begin{aligned} MCC= & {} \frac{tp*tn-fp*fn}{\sqrt{(tp+fp)*(tp+fn)*(tn+fp)*(tn+fn)}} \end{aligned}$$

where true positive (*tp*) represents that both the prediction and actual label was true. True negative (*tn*) represents that both the prediction and actual label was true. In case of false positive (*fp*), the prediction outcome was true but in reality it was false. And finally, false negative (*fn*), the predictions were false but in reality it was true.

### Feature ranking

Feature ranking is a technique to select a subset of input features that are most relevant to the target variable we are trying to predict. The feature selection process is essential as irrelevant and redundant featues may distract the ML algorithms and may result in a lower predictive performance. We used SHapley Additive exPlanations (SHAP) [33] for calculating feature importance of features.

## Results

### Ablation study based on different types of features used in MSLP

To compare the effectiveness of different types of features in developing MSLP, we conducted an ablation study on each of the four types of features. Tables 2, 3, 4, 5, 6, highlight the performance of different ML models developed based on different types of features. We used accuracy as an evaluation metric to identify the best performer. The outcome of the study is discussed below.

For the Cytoplasm class, the best accuracy (82.90%) was obtained when the k-mer features were used. For Nucleus, both the k-mer and PseKNC features demonstrated identical predictive ability in achieving the highest accuracy (86.3%). For the ER, the PseKNC and Z-curve feature was found to be the top-two best representations with over 72% accuracy. Lastly, for Mitochondria, Z-curve feature showed the best performance with an accuracy of 99.30%. These findings sum up as our third contribution in this work as it demonstrates that different sets of features represent the sequences best for localization at different locations.

When we used the combination of all features to identify the predicted subcellular location, CatBoost demonstrated the best performance in three out of five cases. In the other two, RF and XGB came out on top. Hence, it was an ensemble-based method that was the winner for all locations.

**Fig. 5:**
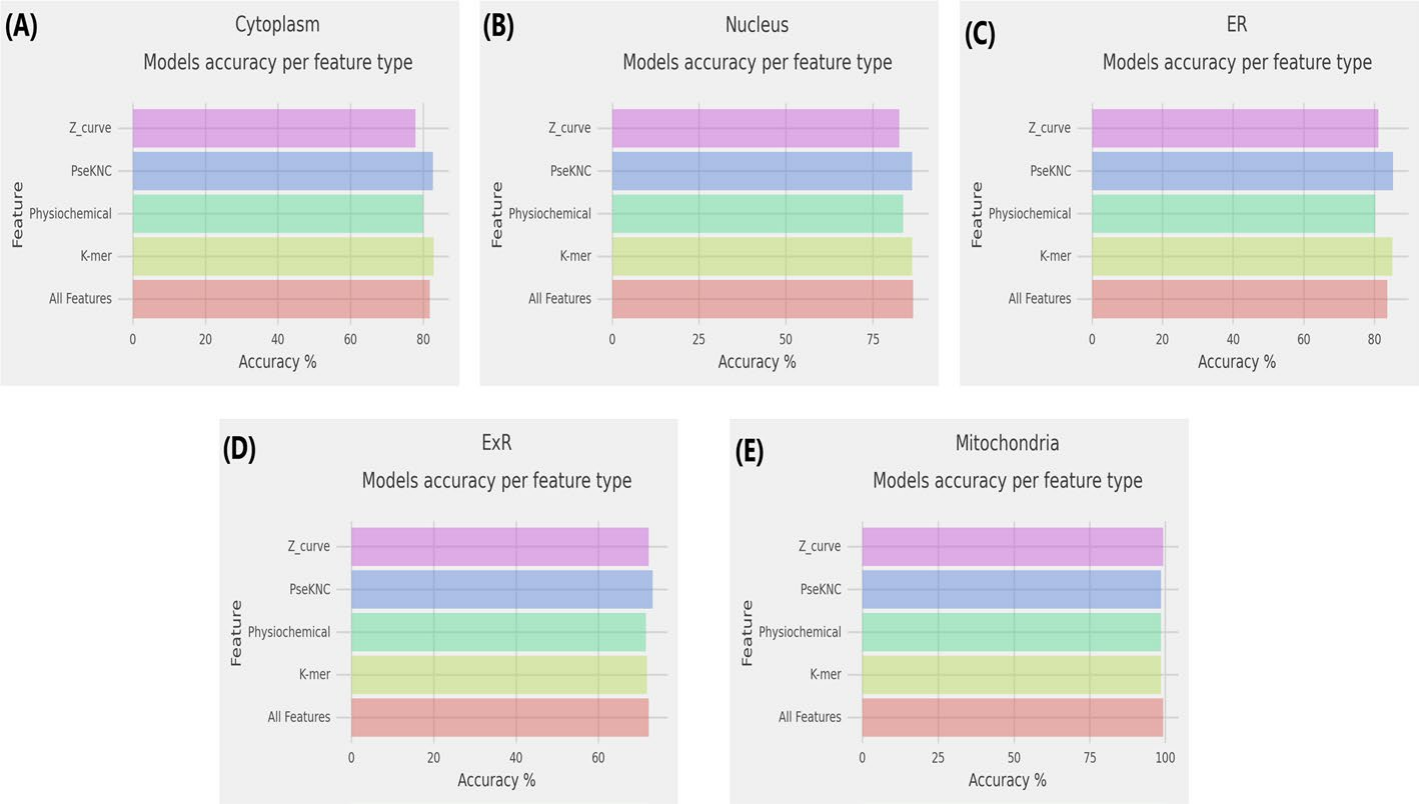
Results on ablation study for mRNA subcellular localiztion prediction of **a** Cytoplasm, **b** Nucleus, **c** ER, **d** ExR, and **e** Mitochondria. We have decided to use accuracy as the evaluation metric

Figure 5 highlights the performance of MSLP on five different subcellular localization prediction for CatBoost model with accuracy as an evaluation metric.

**Table 2:** Results from ablation study—cytoplasm

Cytoplasm (5310)	Metric	GNB	DT	RF	XGB	CatB	SVC
Kmer	Sen	56.70	70.50	82.20	84.00	84.20	85.40
	Spe	72.90	72.40	82.50	81.70	80.80	79.20
	ACC	64.80	71.50	82.30	82.90	82.50	82.30
	Pre	72.90	72.40	82.50	81.70	80.80	79.20
	F1	64.60	71.50	82.30	82.90	82.50	82.30
	MCC	0.3	0.429	0.647	0.657	0.65	0.647
PseKNC	Sen	55.00	73.70	82.10	84.30	84.50	85.20
	Spe	73.90	73.10	82.50	80.30	80.90	79.00
	ACC	64.50	73.40	82.30	82.30	82.70	82.10
	Pre	73.90	73.10	82.50	80.30	80.90	79.00
	F1	64.10	73.40	82.30	82.30	82.70	82.10
	MCC	0.294	0.468	0.646	0.646	0.654	0.643
Physiochemical	Sen	53.80	68.90	81.10	80.40	81.50	79.90
	Spe	74.50	69.50	79.00	77.60	75.40	68.70
	ACC	64.10	69.20	80.00	79.00	78.50	74.30
	Pre	74.50	69.50	79.00	77.60	75.40	68.70
	F1	63.70	69.20	80.00	79.00	78.50	74.30
	MCC	0.289	0.384	0.601	0.58	0.571	0.49
Z-curve	Sen	56.40	67.50	78.80	80.30	79.50	81.60
	Spe	73.20	69.30	76.80	75.40	72.60	70.40
	ACC	64.80	68.40	77.80	77.90	76.00	76.00
	Pre	73.20	69.30	76.80	75.40	72.60	70.40
	F1	64.50	68.40	77.80	77.90	76.00	76.00
	MCC	0.3	0.368	0.557	0.558	0.522	0.524
All features	Sen	55.30	71.10	83.00	83.50	84.40	64.70
	Spe	73.80	72.20	80.60	78.00	78.70	69.40
	ACC	64.50	71.70	81.80	80.70	81.50	67.00
	Pre	73.80	72.20	80.60	78.00	78.70	69.40
	F1	64.20	71.70	81.80	80.70	81.50	67.00
	MCC	0.296	0.433	0.636	0.616	0.632	0.341

**Table 3:** Results from ablation study—nucleus

Nucleus (4855)	Metric	GNB	DT	RF	XGB	CatB	SVC
Kmer	Sen	82.90	75.00	86.30	87.20	85.80	85.00
	Spe	54.40	73.50	86.30	85.30	85.90	86.40
	ACC	68.60	74.30	86.30	86.30	85.80	85.70
	Pre	54.40	73.50	86.30	85.30	85.90	86.40
	F1	68.00	74.30	86.30	86.20	85.80	85.70
	MCC	0.389	0.485	0.726	0.725	0.717	0.714
PseKNC	Sen	83.20	76.10	86.50	86.50	86.30	84.80
	Spe	54.20	72.90	85.90	86.10	85.90	85.90
	ACC	68.70	74.50	86.20	86.30	86.10	85.30
	Pre	54.20	72.90	85.90	86.10	85.90	85.90
	F1	68.00	74.50	86.20	86.30	86.10	85.30
	MCC	0.391	0.49	0.724	0.726	0.722	0.707
Physiochemical	Sen	82.60	77.80	83.70	84.90	84.80	82.10
	Spe	49.80	72.50	83.60	82.30	80.20	75.70
	ACC	66.20	75.10	83.70	83.60	82.50	78.90
	Pre	49.80	72.50	83.60	82.30	80.20	75.70
	F1	65.30	75.10	83.70	83.60	82.50	78.90
	MCC	0.343	0.503	0.674	0.672	0.651	0.579
Z-curve	Sen	80.70	73.90	83.70	85.40	85.10	82.00
	Spe	53.10	71.50	80.60	79.30	80.10	80.40
	ACC	66.90	72.70	82.20	82.30	82.60	81.20
	Pre	53.10	71.50	80.60	79.30	80.10	80.40
	F1	66.30	72.70	82.20	82.30	82.60	81.20
	MCC	0.353	0.454	0.644	0.648	0.653	0.624
All features	Sen	83.90	78.40	86.20	87.50	87.00	79.90
	Spe	51.30	74.20	85.30	85.70	86.20	63.90
	ACC	67.60	76.30	85.70	86.60	86.60	71.90
	Pre	51.30	74.20	85.30	85.70	86.20	63.90
	F1	66.70	76.30	85.70	86.60	86.60	71.70
	MCC	0.373	0.526	0.715	0.732	0.732	0.443

**Table 4:** Results from ablation study for ER

ER (1185)	Metric	GNB	DT	RF	XGB	CatB	SVC
Kmer	Sen	61.60	68.80	79.30	83.10	83.10	87.30
	Spe	70.60	62.60	80.70	80.70	83.60	82.80
	ACC	66.10	65.70	80.00	81.90	83.40	85.10
	Pre	70.60	62.60	80.70	80.70	83.60	82.80
	F1	66.00	65.70	80.00	81.90	83.40	85.00
	MCC	0.323	0.314	0.6	0.638	0.667	0.702
PseKNC	Sen	59.10	65.80	79.70	86.90	85.20	87.80
	Spe	73.10	67.20	84.90	79.00	84.50	82.80
	ACC	66.10	66.50	82.30	82.90	84.80	85.30
	Pre	73.10	67.20	84.90	79.00	84.50	82.80
	F1	65.90	66.50	82.30	82.90	84.80	85.30
	MCC	0.325	0.331	0.647	0.661	0.697	0.706
Physiochemical	Sen	56.50	66.70	77.60	80.20	81.40	83.10
	Spe	73.90	67.20	77.30	80.30	79.00	71.40
	ACC	65.30	66.90	77.50	80.20	80.20	77.30
	Pre	73.90	67.20	77.30	80.30	79.00	71.40
	F1	65.00	66.90	77.50	80.20	80.20	77.20
	MCC	0.31	0.339	0.549	0.604	0.604	0.549
Z-curve	Sen	58.60	64.60	75.10	81.90	81.90	85.20
	Spe	70.20	65.10	81.90	76.10	80.30	76.10
	ACC	64.40	64.80	78.50	78.90	81.10	80.60
	Pre	70.20	65.10	81.90	76.10	80.30	76.10
	F1	64.30	64.80	78.50	78.90	81.10	80.60
	MCC	0.29	0.297	0.572	0.58	0.621	0.615
All features	Sen	58.20	73.00	79.70	85.70	86.50	77.20
	Spe	73.10	71.00	78.20	78.60	80.70	71.40
	ACC	65.70	72.00	78.90	82.10	83.60	74.30
	Pre	73.10	71.00	78.20	78.60	80.70	71.40
	F1	65.50	72.00	78.90	82.10	83.60	74.30
	MCC	0.317	0.44	0.579	0.644	0.673	0.487

**Table 5:** Results from ablation study for ExR

ExR (710)	Metric	GNB	DT	RF	XGB	CatB	SVC
Kmer	Sen	52.80	49.30	71.80	69.70	74.60	73.20
	Spe	76.10	60.60	69.00	71.10	69.00	66.90
	ACC	64.40	54.90	70.40	70.40	71.80	70.10
	Pre	76.10	60.60	69.00	71.10	69.00	66.90
	F1	63.90	54.80	70.40	70.40	71.80	70.00
	MCC	0.297	0.099	0.409	0.408	0.437	0.402
PseKNC	Sen	54.20	55.60	73.20	72.50	70.40	73.20
	Spe	74.60	64.10	73.20	73.20	69.70	67.60
	ACC	64.40	59.90	73.20	72.90	70.10	70.40
	Pre	74.60	64.10	73.20	73.20	69.70	67.60
	F1	64.10	59.80	73.20	72.90	70.10	70.40
	MCC	0.295	0.198	0.465	0.458	0.401	0.409
Physiochemical	Sen	69.70	64.10	71.10	69.70	77.50	78.20
	Spe	49.30	60.60	63.40	65.50	65.50	58.50
	ACC	59.50	62.30	67.30	67.60	71.50	68.30
	Pre	49.30	60.60	63.40	65.50	65.50	58.50
	F1	59.10	62.30	67.20	67.60	71.40	68.00
	MCC	0.194	0.247	0.346	0.352	0.433	0.374
Z-curve	Sen	52.10	52.80	66.90	74.60	78.20	76.10
	Spe	68.30	52.80	60.60	64.10	66.20	62.70
	ACC	60.20	52.80	63.70	69.40	72.20	69.40
	Pre	68.30	52.80	60.60	64.10	66.20	62.70
	F1	59.90	52.80	63.70	69.30	72.10	69.20
	MCC	0.207	0.056	0.275	0.39	0.447	0.391
All features	Sen	68.30	58.50	78.90	73.20	75.40	76.10
	Spe	62.70	63.40	62.70	70.40	69.00	40.80
	ACC	65.50	60.90	70.80	71.80	72.20	58.50
	Pre	62.70	63.40	62.70	70.40	69.00	40.80
	F1	65.50	60.90	70.60	71.80	72.20	57.10
	MCC	0.31	0.219	0.421	0.437	0.445	0.181

**Table 6:** Results from ablation study for mitochondria

Mitochondria (350)	Metric	GNB	DT	RF	XGB	CatB	SVC
Kmer	Sen	98.60	90.00	98.60	98.60	98.60	98.60
	Spe	85.70	94.30	98.60	98.60	98.60	97.10
	ACC	92.10	92.10	98.60	98.60	98.60	97.90
	Pre	85.70	94.30	98.60	98.60	98.60	97.10
	F1	92.10	92.10	98.60	98.60	98.60	97.90
	MCC	0.85	0.844	0.971	0.971	0.971	0.957
PseKNC	Sen	98.60	88.60	98.60	98.60	98.60	98.60
	Spe	87.10	90.00	98.60	98.60	98.60	97.10
	ACC	92.90	89.30	98.60	98.60	98.60	97.90
	Pre	87.10	90.00	98.60	98.60	98.60	97.10
	F1	92.80	89.30	98.60	98.60	98.60	97.90
	MCC	0.863	0.786	0.971	0.971	0.971	0.957
Physiochemical	Sen	92.90	90.00	95.70	97.10	98.60	98.60
	Spe	78.60	95.70	98.60	98.60	98.60	98.60
	ACC	85.70	92.90	97.10	97.90	98.60	98.60
	Pre	78.60	95.70	98.60	98.60	98.60	98.60
	F1	85.60	92.90	97.10	97.90	98.60	98.60
	MCC	0.722	0.859	0.943	0.957	0.971	0.971
Z-curve	Sen	98.60	98.60	98.60	98.60	98.60	98.60
	Spe	97.10	97.10	98.60	100.00	100.00	100.00
	ACC	97.90	97.90	98.60	99.30	99.30	99.30
	Pre	97.10	97.10	98.60	100.00	100.00	100.00
	F1	97.90	97.90	98.60	99.30	99.30	99.30
	MCC	0.957	0.957	0.971	0.986	0.986	0.986
All features	Sen	95.70	91.40	97.10	98.60	98.60	94.30
	Spe	80.00	97.10	98.60	100.00	98.60	95.70
	ACC	87.90	94.30	97.90	99.30	98.60	95.00
	Pre	80.00	97.10	98.60	100.00	98.60	95.70
	F1	87.80	94.30	97.90	99.30	98.60	95.00
	MCC	0.767	0.887	0.957	0.986	0.971	0.90

### Comparison of MSLP against other existing methods

For the comparison of MSLP against other methods, we used the results from the first experiment configuration. We considered the same dataset and locations, and compared it against three other existing methods. Based on our results we can observe that our method was able to outperform other methods in cross-validation (Table 7) in all metrics in three out of five locations, and in a majority of the metrics in another. It is worthy to mention that in mLoc-mRNA, the authors did not use any data for ExR prediction. Therefore, we were not able to compare the performance for ExR against mLoc-mRNA. For iLoc-mRNA, ExR was not considered as a location, as a result we were not able to compare the performance for ExR against iLoc-mRNA. Moreover in iLoc-mRNA Cytoplasm was combined with Cytosol as C1, Mitochondria and Nucleus were combined with Exosome and Dendrites as C4. Therefore, we can not directly compare the performance of our method against iLoc-mRNA.

On the test set TEST-01 (Table 8), our method outperformed the other methods in a majority of the metrics in three out of five locations. MSLP outperformed mRNALoc in terms of Sn, and Acc for nucleus. For cytoplasm, MSLP outperformed mRNALoc in terms of Sp, and Acc. For this two locations (cytoplasm and nucleus) with relatively higher number of samples, MSLP outperformed its peers in Acc with big margin. For ER, MSLP achieved much better performance in terms of Sn at the cost of Sp. For ExR, MSLP outperformed mRNALoc with huge margin for Sp and Acc. For Mitochondria, MSLP achieved Sn, Sp and Acc of above 90%. All these results clearly highlight the superior performance of MSLP on the TEST-01 dataset.

For the comparison of MSLP for other locations, introduced in [21], we considered the independent dataset IDS-01 and IDS-02 that was proposed in the same. Based on our results we can observe that our method MSLP was able to outperform mLoc-mRNA in both IDS-01 and IDS-02 for posterior, pseudopodia and exosomes (Table 9) when sensitivy and specificity are considered. For ribosomes and cytosol, we were able to outperform mLoc-mRNA in IDS-02 when accuracy is compared. Our model was not able to outperform mLoc-mRNA for cytosol and ribosome solely on IDS-01.

**Table 7:** Performance of MSLP and other methods on cross validation for five locations

Location	Metric	mRNALoc	mLoc-mRNA	iLoc-mRNA	MSLP
Cytoplasm	Sn	66.69	73.24	Com	**84.60**
	Sp	67.41	68.51	Com	**81.60**
	Acc	67.10	70.87	Com	**83.10**
Nucleus	Sn	74.17	72.89	Com	**86.80**
	Sp	73.22	73.99	Com	**86.00**
	Acc	73.59	73.44	Com	**86.40**
ER (C3 for iLoc-mRNA)	Sn	74.09	63.04	**89.72**	80.80
	Sp	75.49	73.68	**97.56**	78.70
	Acc	75.36	68.36	NR	79.70
ExR	Sn	62.67	–	–	**70.10**
	Sp	65.34	–	–	**70.20**
	Acc	65.20	–	–	**70.10**
Mitochondria	Sn	96.28	98.53	C	**97.80**
	Sp	**99.80**	91.70	Com	98.10
	Acc	99.70	96.46	Com	**97.90**

For our method MSLP, we mentioned the results from the CatBoost model.–: Location was not considered in the literature. Com: Location was combined with other locations, therefore not comparable; NR: Not reported. Bold numbers highlight the best value of performance metrics

**Table 8:** Performance of MSLP and other Tools on TEST-01 for five locations

Location (no of sequence)	Metric	mRNALoc	mRNALocater	MSLP
Cytoplasm (1066)	Sn	73.26	**79.64**	61.80
	Sp	58.06	–	**77.30**
	Acc	64.55	–	**70.30**
Nucleus (976)	Sn	50.20	26.13	**71.70**
	Sp	**81.62**	–	75.00
	Acc	69.35	–	**73.70**
ER (241)	Sn	75.10	09.13	**85.10**
	Sp	**68.60**	–	61.40
	Acc	**69.23**	–	63.70
ExR (145)	Sn	81.38	**95.86**	78.60
	Sp	56.67	–	**75.50**
	Acc	58.10	–	**75.70**
Mitochondria (71)	Sn	87.32	83.10	**98.60**
	Sp	**97.16**	–	90.30
	Acc	**96.88**	–	90.50

For our method MSLP, we mentioned the results from the CatBoost model. ”–”:mRNALocater did not consider any negative set, so it is not comparable. Bold numbers highlight the best value of performance metrics

**Table 9:** Performance of MSLP and mLoc-mRNA based on independent datasets (IDS)

Location		IDS-01		IDS-02	
	Metric	MSLP	mLoc-mRNA	MSLP	mLoc-mRNA
Cytosol	Sn	**70.60**	64.17	86.80	89.78
	Sp	62.40	72.98	67.30	71.73
	Acc	66.50	71.37	**77.00**	75.91
Pseudopodium	Sn	**66.70**	55.56	89.90	93.67
	Sp	**69.40**	65.72	68.40	69.02
	Acc	**68.10**	65.53	**79.10**	69.45
Posterior	Sn	100.00	100.00	100.00	100.00
	Sp	**96.80**	93.50	**99.20**	92.97
	Acc	**98.40**	93.60	**99.60**	93.16
Exosome	Sn	**71.40**	66.43	85.90	88.65
	Sp	**78.60**	73.50	74.10	77.65
	Acc	**75.00**	72.99	**80.00**	78.10
Ribosome	Sn	66.30	66.34	87.00	91.48
	Sp	65.40	74.76	69.40	73.73
	Acc	65.80	73.45	**78.20**	76.89

Bold numbers highlight the best value of performance metrics

### Important features proposed by MSLP on different subcellular locations

**Fig. 6:**
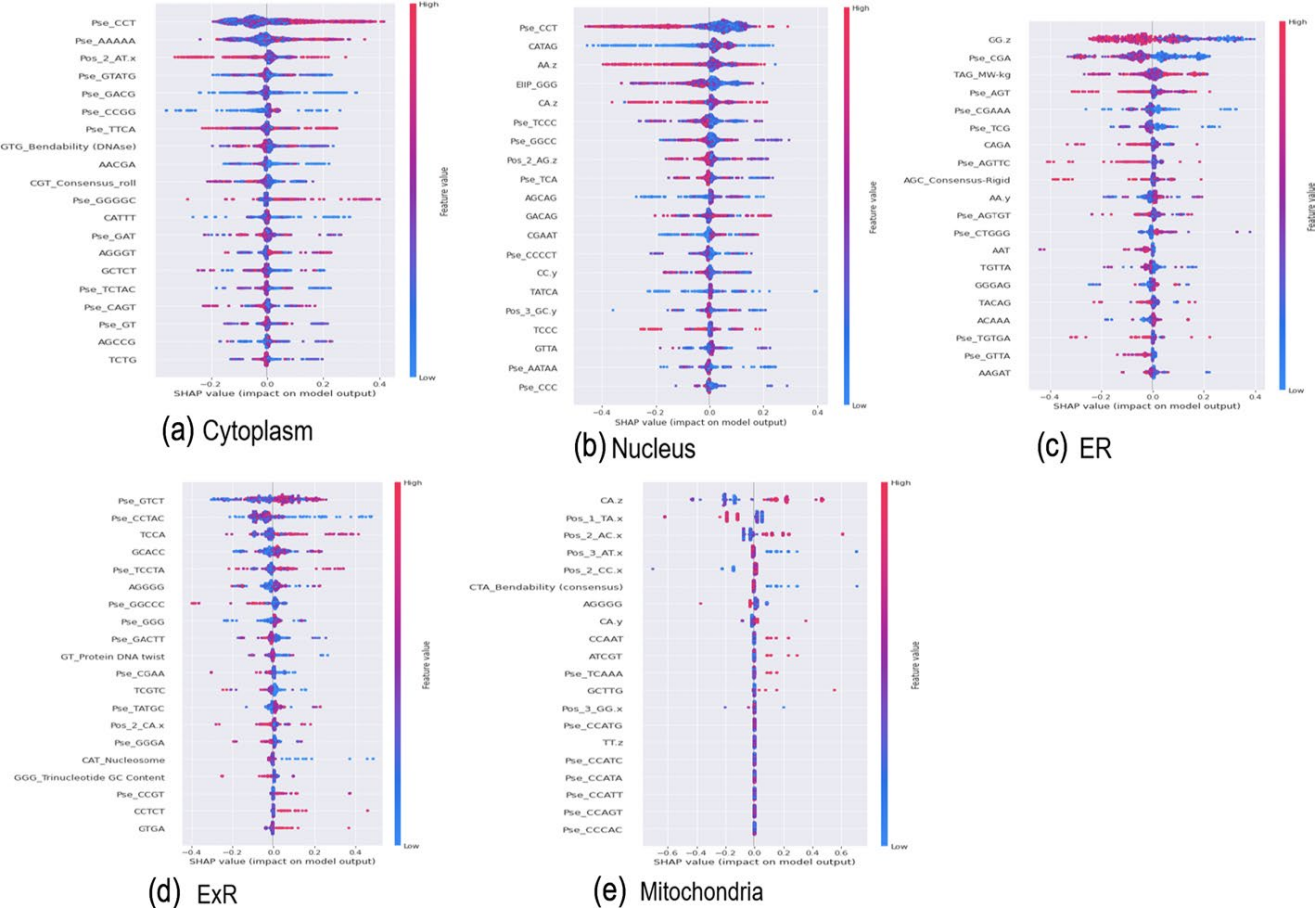
SHAP Analysis for top 10 features for different sucellular locations

Unlike the traditional statistical analysis, complex models built upon ML techniques can be more challenging to explain and justify for human users. Therefore, we used SHAP (SHapley Additive exPlanation) to get insights into the important features for each sub localization and explain the model’s predictions. The actual selected features for all subcellular localizations are all listed and described in Additional file 1: File S3. Figure 6 highlights the most important features proposed by SHAP for different subcellular localizations.

For cytoplasm, nucleus and ER, k-mer and PseKNC related features were more dominant than other features (Fig. 6a, b, c). Interestingly Pse_KNC for trinucleotide for CCT (Pse_CCT) was the top ranked feature for both cytoplasm and nucleus prediction, but the value of Pse_CCT was much higher for cytoplasm prediction (Fig. 6a) and opposite trend was observed for nucleus prediction (Fig. 6b). Z-curve transformation of AT in x-axis, bendability (DNase) of GTG and consensus roll of CGT were among the top ten features for predicting cytoplasm localized mRNAs (Fig. 6a). On the other hand, EIIP of GGG, Z-curve transformation of AA, CA and AG in the z-axis were among the top ten features for predicting nucleus localized mRNAs (Fig. 6b). For ER, Z-curve transformation of GG in z-axis was the top ranked feature showing relatively lower values in mRNA localized in ER compared to other locations (Fig. 6c).

For mitochondria, Z-curve representation of sequence for di-nucleotides were the top ranked five features (Fig. 6e). Among them, phase-independent representation of CA in the z-axis was much higher in the positive class (mitochondria) compared to the negative class. Phase-dependent Z-cure representation of di-nucleotides AC, AT, CC and TA in the x-axis had opposite trends in the positive and negative class, moving them towards the top contributors in mitochondria localization prediction task. Moreover, bendability of tri-nucleotide CTA was much lower in the mitochondria localized mRNAs compared to mRNAs from other locations. Overall, this highlights the importance of physicochemical properties and 3D representation of sequences in this prediction task.

## Discussions

In this article, we propose MSLP, a machine learning based approach to predict mRNA subcellular localization covering ten locations based on the dataset collected from existing literature. To develop MSLP, we used standard K-mer features, PseKNC, physicochemical Properties of mRNA like PseEIIP, DPCP and TPCP; and the Z-curve parameters for phase-specific and phase-independent trinucleotide frequencies (48 bit and 144 bit). For the classification problem, we considered the OvR strategy for the mRNA subcellular localization prediction due to a few reasons: (i) previous works [18, 19, 21–23] highlighted in Table 1 use the OvR approach; using the same in ours enables us to compare our result with those, and (ii) the classifier’s task in a multi-class classification setting is considerable more complex than the multiple OvR tasks as the sole classifier in the former has to learn five hyper-planes to learn the data distribution. On the other hand, the OvR classifiers have a comparatively simpler task where each has to learn a single hyper-plane to distinguish between the designated class and the rest. Since each class is intended for one and one classifier only, it provides more insights into the class by inspecting its corresponding classifier. Based on our methods, we concluded that ensemble based methods e.g., RF, XGB or CatBoost were more effective than non-ensemble classifiers such as DT, SVC or GNB. Feature ranking methods supported our results by revealing the more important features for the prediction task. Based on our results, there was no specific set of features that were dominant across all subcellular localizations of mRNA. For cytoplasm, nucleus and ER, we observed the impact of k-mer and PseKNC were more dominant than physicochemical properties (Tables 2, 3 and 4). But for ExR and mitochondria we observed higher contributions from physicochemical properties and Z-curve, emphasizing the importance of varieties and types of features for this problem (Tables 5 and 6). Comparative analysis of the proposed MSLP against other methods for multiple benchmark datasets highlighted the superiority of the proposed approach in our study (Tables 8 and 9).

Moreover, we noticed that different research groups are considering different cellular sublocations as a part of their analyses, which makes it difficult to establish a single dataset as a benchmark and compare against it. Initially CeFra-Seq and APEX-RIP based dataset was used in [1] for this purpose. Then other groups started to focus on using the RNALocate database [20] as the gold standard dataset for this problem. Majority of the published work focused on five locations namely cytoplasm, nucleus, ER, ExR, mitochondria (Table 1). Recently, Meher et al. [21] proposed a new dataset with nine subcellular localizations of mRNA. In our study, we combined all these datasets covering ten subcellular localizations and tested MSLP on multiple independent datasets to evaluate its performance.

Our study has some limitations that need to be pointed out. Like the previous studies [18, 19, 21, 22], we considered only mRNAs which were localized only in one subcellular location. Hence, this limits our findings to be applicable to a subset of mRNAs. Though we have highlighted in Table 1 that many other studies have considered mRNAs coming from one location which clearly indicates the challenges of predicting mRNAs from multiple locations. Recently DM3Loc [34] method is proposed to predict sub-cellular localization considering the multi-label nature of mRNA localization. We will consider the similar approach as part of our future studies.

We believe the mRNA subcellular localization problem will require more attention from the RNA community to standardize the benchmark datasets for different subcellular locations. In future, we plan to investigate the localization problem for mRNAs with more than one location for the multi-label classification problem. We believe this will provide a more realistic picture of the landscape of mRNA localization. Our model will complement the existing prediction methods for mRNA subcellular localization prediction and support the wet lab validations.

## Conclusion

Different cellular compartments are required for the biological function of RNA biomolecules in eukaryotic cells. The subcellular localization of the mRNAs is currently determined using labor-intensive, expensive, and time-consuming wet-lab procedures. We considered an OvR classification approach to tackle the multi-class classification problem for the mRNA subcellular localization prediction task. We evaluated our method, MSLP on multiple benchmark datasets covering ten subcellular locations for mRNA. We propose a novel combination of features representing DNA sequence of mRNA using k-mer, pseudo nucleotide composition, physicochemical properties, and 3D representation of sequence in Z-curve transformation to predict mRNA localization. We showed that k-mer and PseKNC were more dominant than other features for predicting cytoplasm, nucleus, and ER. But physicochemical properties and Z-curve-based features were considered the dominant feature set for ExR and mitochondria detection. We plan to investigate the localization problem further using multi-label classification methods and deep neural network (deep learning)-based techniques in future. We are also planning to investigate the localization problem from the non-coding domain. We believe this will provide a complete picture of the localization landscape by covering the significant groups of RNA, i.e., coding and non-coding RNAs.

## Supplementary information

**Additional file 1: File S1.** List of physicochemical properties related to DPCP and TPCP. **File S2.** Name of the features and their mapping. **File S3.** Selected top features and their importance based on SHAP analysis for locations.

## Data Availability

Source code and data is shared on GitHub at: https://github.com/smusleh/MSLP.
