# Correction: MSLP: mRNA subcellular localization predictor based on machine learning techniques

**DOI:** 10.1186/s12859-023-05276-2

**Published:** 2023-04-18

**Authors:** Saleh Musleh, Mohammad Tariqul Islam, Rizwan Qureshi, Nehad M. Alajez, Tanvir Alam

**Affiliations:** 1 College of Science and Engineering, Hamad Bin Khalifa University, Doha, Qatar; 2 Computer Science Department, Southern Connecticut State University, New Haven, CT USA; 3 Translational Cancer and Immunity Center (TCIC), Qatar Biomedical Research Institute (QBRI), Hamad Bin Khalifa University, Doha, Qatar; 4 College of Health and Life Sciences, Hamad Bin Khalifa University, Doha, Qatar

**Correction: BMC Bioinformatics (2023) 24:109** 10.1186/s12859-023-05232-0

Following publication of the original article [1], the authors identified an error in the author name of Nehad M. Alajez.

The incorrect author name is: Nihad Alajez

The correct author name is: Nehad M. Alajez

The author group has been updated above and the original article [1] has been corrected.

## References

[1] Musleh (2023). MSLP: mRNA subcellular localization predictor based on machine learning techniques. BMC Bioinform.

