# Supplementary material for: MSLP: mRNA subcellular localization predictor based on machine learning techniques
Source: BMC Bioinformatics. 2023 Mar 22;24:109. doi: 10.1186/s12859-023-05232-0 (PMC10035125; doi:10.1186/s12859-023-05232-0)
Supplement: Supplementary file 1 — Additional file 1: File S1. List of physicochemical properties related to DPCP and TPCP. File S2. Name of the features and their mapping. File S3. Selected top features and their importance based on SHAP analysis for locations. [file 12859_2023_5232_MOESM1_ESM.pdf]

Additional File S1: List of physicochemical properties related to DPCP and TPCP.

Physicochemical properties for DPCP

Base stacking	Protein induced deformability	B-DNA twist	Propeller twist	Duplex stability:(freeenergy)
Duplex tability(disruptenergy)	Protein DNA twist	Stabilising energy of Z-DNA	Aida_BA_transition	Breslauer_dS
Electron_interaction	Hartman_trans_free_energy	Lisser_BZ_transition	Polar_interaction	SantaLucia_dG
Sarai_flexibility	Stability	Stacking_energy	Sugimoto_dS	Watson-Crick_interaction
Twist	Shift	Slide	Rise	Twist stiffness
Tilt stiffness	Shift_rise	Twist_shift	Enthalpy1	Twist_twist
Shift2	Tilt3	Tilt1	Slide (DNA-protein complex) 1	Tilt_shift
Twist_tilt	Roll_rise	Stacking energy	Stacking energy1	Propeller Twist
Roll11	Rise (DNA-protein complex)	Roll2	Roll3	Roll1
Slide_slide	Enthalpy	Shift_shift	Flexibility_slide	Minor Groove Distance
Rise (DNA-protein complex)1	Roll (DNA-protein complex)1	Entropy	Cytosine content	Major Groove Distance
Twist (DNA-protein complex)	Purine (AG) content	Tilt_slide	Major Groove Width	Major Groove Depth
Free energy6	Free energy7	Free energy4	Free energy3	Free energy1
Twist_roll	Flexibility_shift	Shift (DNA-protein complex) 1	Thymine content	Tip
Keto (GT) content	Roll stiffness	Entropy1	Roll_slide	Slide (DNA-protein complex)
Twist2	Twist5	Twist4	Tilt (DNA-protein complex)1	Twist_slide
Minor Groove Depth	Persistance Length	Rise3	Shift stiffness	Slide3
Slide2	Slide1	Rise1	Rise stiffness	Mobility to bend towards
Dinucleotide GC Content	A-philicity	Wedge	DNA denaturation	Bending stiffness
Free energy5	Breslauer_dG	Breslauer_dH	Shift (DNA-	Helix-Coil_transition
Ivanov_BA_transition	Slide_rise	SantaLucia_dH	SantaLucia_dS	Minor Groove Width

Sugimoto_dG	Sugimoto_dH	Twist1	Tilt	Roll
Twist7	Clash Strength	Roll_roll	Roll (DNA-protein	Adenine content
Direction	Probability contacting	Roll_shift	Shift_slide	Shift1
Tilt4	Tilt2	Free energy8	Twist (DNA-	Tilt_rise
Free energy2	Stacking energy2	Stacking energy3	Rise_rise	Tilt_tilt
Roll4	Tilt_roll	Minor Groove Size	GC content	Inclination
Slide stiffness	Melting Temperature1	Twist3	Tilt (DNA-protein	Guanine content
Twist6	Major Groove Size	Twist_rise	Rise2	Melting Temperature
Free energy	Mobility to bend towards	Bend		

Physicochemical properties for TPCP

Dnase I	Bendability (DNAse)	Bendability	Trinucleotide GC
Nucleosome positioning	Consensus_roll	Consensus-Rigid	Dnase I-Rigid
MW-Daltons	MW-kg	Nucleosome	Nucleosome-

Additional File S2: Name of the features and their mapping.

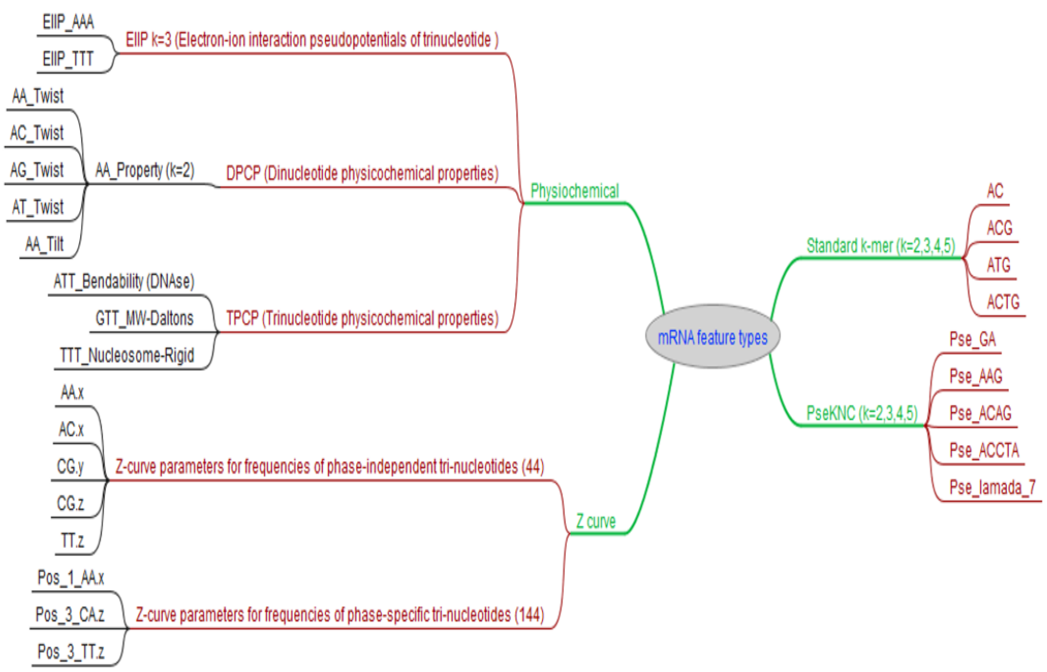

Additional File S3: Selected top features and their importance based on SHAP analysis for locations

Cytoplasm

col_name	feature_importance_vals
Pse_CCT	206.673056
Pse_AAAAA	116.444305
Pos_2_AT.x	74.121072
Pse_GTATG	48.02352
Pse_GACG	46.29276
Pse_CCGG	43.248298
Pse_TTCA	42.917792
GTG_Bendability (DNAse)	41.368288
AACGA	39.938774
CGT_Consensus_roll	38.964102
Pse_GGGGC	35.182722
CATTT	28.860437
Pse_GAT	27.656036
AGGGT	26.523641
GCTCT	24.929051
Pse_TCTAC	23.68738
Pse_CAGT	23.544006
Pse_GT	21.557911
AGCCG	18.609311
TCTG	18.351851

Nucleus

col_name	feature_importance_vals
Pse_CCT	187.077578
CATAG	103.88836
AA.z	92.803388
EIIP_GGG	87.853341
CA.z	6.833876
Pse_TCCC	50.697388
Pse_GGCC	41.466302
Pos_2_AG.z	33.834258
Pse_TCA	30.258604
AGCAG	29.527333
GACAG	29.239678
CGAAT	26.264532
Pse_CCCCT	25.990881
CC.y	25.816443
TATCA	21.579559
Pos_3_GC.y	20.970483
TCCC	20.635664
GTTA	19.271744
Pse_AATAA	19.06901
Pse_CCC	17.802511

ER

col_name	feature_importance_vals
GG.z	62.424883
Pse_CGA	46.826436
TAG_MW-kg	25.152063
Pse_AGT	18.673658
Pse_CGAAA	14.414912
Pse_TCG	13.482978
CAGA	11.115082
Pse_AGTTC	10.804254
AGC_Consensus-Rigid	10.618663
AA.y	10.461962
Pse_AGTGT	9.715807
Pse_CTGGG	9.417216
AAT	9.14116
TGTTA	7.662036
GGGAG	7.484691
TACAG	6.898512
ACAAA	6.744645
Pse_TGTGA	6.548677
Pse_GTTA	6.360968
AAGAT	5.77078

EXR

col_name	feature_importance_vals
Pse_GTCT	28.846373
Pse_CCTAC	24.563183
TCCA	17.138336
GCACC	13.030264
Pse_TCCTA	12.843472
AGGGG	10.559118
Pse_GGCCC	8.395368
Pse_GGG	7.892994
Pse_GACTT	7.254099
GT_Protein DNA twist	5.718517
Pse_CGAA	5.530338
TCGTC	5.309657
Pse_TATGC	5.038264
Pos_2_CA.x	5.01998
Pse_GGGA	4.821176
CAT_Nucleosome	4.737092
GGG_Trinucleotide GC Content	4.697096
Pse_CCGT	4.304871
CCTCT	3.969292
GTGA	3.670255

Mitochondria

col_name	feature_importance_vals
CA.z	29.34091
Pos_1_TA.x	14.02338
Pos_2_AC.x	12.214785
Pos_3_AT.x	7.009193
Pos_2_CC.x	5.582243
CTA_Bendability (consensus)	3.881014
AGGGG	3.022339
CA.y	2.27599
CCAAT	1.780037
ATCGT	1.251607
Pse_TCAAA	1.048577
GCTTG	1.016545
Pos_3_GG.x	0.564205
GG_Minor Groove Distance	0
TT_Minor Groove Distance	0
TG_Minor Groove Distance	0
CT_Flexibility_slide	0
CG_Flexibility_slide	0
CC_Flexibility_slide	0
CA_Flexibility_slide	0

Pseudopodium

col_name	feature_importance_vals
PseKNC_lamada_6	13.805925
PseKNC_CAGAG	6.363826
GCGCA	5.822396
Pos_2_AAy	3.624833
PseKNC_GGTAT	3.404801
PseKNC_CGTGG	3.32124
PseKNC_AAAAA	3.260308
TAAC	2.892381
Pos_3_CCz	2.695837
PseKNC_AGAAC	2.267244
CGTA	2.091755
PseKNC_TTCCT	2.000825
PseKNC_TCAG	1.996316
CCACG	1.164365
GATA	1.142898
ATTAG	1.062658
AATCA	1.05816
GGCGT	0.970909
PseKNC_TTGAC	0.851616
Pos_2_CGy	0.744699

Exosome

col_name	feature_importance_vals
GCGA	22.479522
CTx	21.370833
PseKNC_CTGAC	15.862419
PseKNC_ACTGT	15.024765
PseKNC_ACACA	10.369668
GTy	9.329866
CGAGC	9.075361
PseKNC_GGCAA	7.670002
GACAC	6.441558
PseKNC_CATGC	5.773664
PseKNC_TATGC	5.754335
GTC_DnaseIRigid	5.700318
PseKNC_TCCT	5.456513
CCGGA	5.241851
PseKNC_TATCA	5.085149
PseKNC_TACGT	5.008218
Pos_3_TGz	4.584346
CTCT	4.530319
GCC_DnaseI	4.192017
PseKNC_CCTTT	3.86548

Ribosome

col_name	feature_importance_vals
CGz	73.71227
AAAAA	43.770771
AC_MinorGrooveDistance	35.896707
GG_Rise2	22.665376
PseKNC_ACGAC	18.77651
AC_Freeenergy8	15.881936
GGGC	14.824248
TGTG	14.383809
PseKNC_ATAG	12.964419
Pos_2_TTz	12.424472
AC_Twist_roll	12.124697
GACTA	9.875691
PseKNC_GGGAG	9.349708
CA_Shift1	9.248852
TTGC	8.904275
GG_StabilisingenergyofZDNA	8.615998
PseKNC_GTAC	8.251039
PseKNC_GCGCC	7.655031
TCACT	7.579636
PseKNC_AAAAA	7.407486

Cytosol	
col_name	feature_importance_vals
PseKNC_AAAAA	85.929442
TG_Roll	42.531932
CCTCC	29.843055
TGGT	27.115962
GT_Roll1	19.03369
PseKNC_CGAA	18.454454
CGAA	15.675292
TCy	13.237076
PseKNC_GCCCC	13.16231
PseKNC_TTTCT	12.597915
GGTGT	11.697577
AGCTA	11.124263
TCT_Nucleosomepositioning	10.832596
PseKNC_CCTCT	10.282507
GACGA	9.901289
GGAAT	9.487927
TCCC	9.417518
PseKNC_GCATA	8.450371
ATx	8.099528
GGCTG	8.022591

Posterior	
**col_name**	**feature_importance_vals**
CGA_Nucleosomepositioning	25.866967
PseKNC_CCGGG	4.499514
PseKNC_CTTTT	1.629831
Pos_1_ATz	1.279826
CTCGT	1.208085
AC_MinorGrooveSize	0.961555
